# Beyond smoke: culturable airborne bacteria associated with informal waste burning in peri-urban South Africa

**DOI:** 10.3389/fpubh.2026.1965626

**Published:** 2026-10-07

**Authors:** Boitemogelo O. Kwakwa, Shalin Bidassey-Manilal, Natasha Naidoo, Isaac T. Rampedi

**Affiliations:** 1 Department of Geography, Environmental Management and Energy Studies, University of Johannesburg, Johannesburg, South Africa; 2 Department of Environmental Health, University of Johannesburg, Johannesburg, South Africa; 3 South African Medical Research Council, South Africa

**Keywords:** air pollution, bioaerosols, culturable airborne bacteria, environmental exposure, informal waste burning, South Africa, waste management

## Abstract

**Introduction:**

Informal waste burning is an established source of particulate matter and hazardous combustion emissions in many low- and middle-income settings, yet its potential contribution to biological airborne exposure remains poorly characterized. This study investigated culturable airborne bacteria in active informal waste-burning environments in Hammanskraal, South Africa, and examined their spatial variability and potential relevance to environmental exposure.

**Methods:**

A cross-sectional exploratory field investigation was conducted at three active informal waste-burning sites and one non-burning comparison site. Active air samples were collected using a Sampl’Air Pro impactor onto Tryptic Soy Agar at approximate distances of 25, 50 and 100 m from visible combustion areas. Culturable airborne bacterial concentrations were quantified as colony-forming units per cubic meter (CFU/m^3^), and representative cultured isolates were characterized using the Vitek Compact 2 microbial identification system. Structured field observations documented combustion intensity, waste characteristics, smoke-plume behavior and local environmental conditions.

**Results:**

The highest culturable airborne bacterial concentrations occurred at active waste-burning sites, although concentrations varied substantially among sites. Burn-site concentrations ranged from 91 CFU/m^3^ to above the analytical upper quantification limit (>1,259 CFU/m^3^), compared with 35–260 CFU/m^3^ at the comparison site. Sampling distance was weakly associated with bacterial concentration (Spearman’s ρ = −0.14, *p* = 0.701; exploratory linear regression R^2^ = 0.02). Thirty-nine representative isolates were characterized. Gram-positive bacilli constituted the largest phenotypic grouping, while Sphingomonas paucimobilis, Pantoea spp. and several other environmentally associated or opportunistic taxa were recovered across the sampled environments.

**Discussion:**

Active informal waste-burning environments contained viable culturable airborne bacteria, with markedly elevated concentrations observed at some burning sites relative to the non-burning comparison environment. Distance from active combustion alone did not explain the observed spatial variability; site-specific factors including combustion intensity, plume behavior, environmental disturbance and local meteorology may influence short-term bioaerosol occurrence and warrant investigation using more highly replicated designs. These findings identify viable culturable bacteria as an under-characterized component of informal waste-burning environments and provide a basis for future integrated studies of environmental occurrence, human exposure and potential health relevance.

## 1 Introduction

Air pollution remains one of the major environmental determinants of disease and premature mortality worldwide, with exposure to particulate matter and combustion-derived pollutants contributing substantially to respiratory, cardiovascular and other non-communicable diseases (1, 2). Air quality management has consequently focused predominantly on chemical pollutants, particularly particulate matter (PM₂.₅ and PM₁₀), nitrogen oxides (NO_x_), sulfur dioxide (SO₂), ozone (O₃) and other combustion-derived contaminants known to adversely affect human health (1, 3). This scientific and regulatory focus is essential, but it does not encompass the full complexity of material present within polluted air.

Numerous atmospheric bioaerosol investigations have focused on urban background air, agricultural systems, biomass burning, landfill operations and wastewater treatment facilities (4–6). On the other hand, comparatively little attention has been directed towards informal waste burning, despite its widespread occurrence in many low- and middle-income countries (LMICs) and its recognized contribution to ambient air pollution (7, 8). Informal waste burning is common in many LMICs because inadequate waste collection, illegal dumping, limited formal waste-management infrastructure and rapid urbanization create conditions in which municipal waste is burned in open environments. Open waste burning is an established source of particulate matter, trace gases, persistent organic pollutants and other hazardous combustion products (9). However, informal burn sites may also contain substantial microbial reservoirs, including decomposing food waste, vegetation, soil-contaminated materials, sanitary waste, textiles and mixed household refuse—all of which may influence the composition and dispersal of emitted particulate matter and associated bioaerosols while simultaneously creating harsh environmental conditions that selectively favor the persistence of stress-tolerant taxa (10, 11).

These materials are frequently disturbed through dumping, waste sorting, waste picking, pedestrian movement, vehicular traffic and wind-driven resuspension. Active combustion may introduce additional physical processes including thermal uplift, turbulent airflow and smoke-plume transport. Importantly, combustion should not be assumed to generate microorganisms. A more plausible environmental mechanism is that combustion and associated disturbance may influence the mobilization and atmospheric dispersion of microorganisms already present within waste materials and surrounding environmental reservoirs.

Atmospheric particulate matter carries the potential to transport biological material, including bacteria, fungi, viruses and associated cellular components (4, 12, 13). Evidence from combustion- and particulate-related environments, including biomass burning and dust resuspension systems indicates that viable microorganisms can become aerosolized and transported under combustion-affected conditions (14, 15). Collectively referred to as bioaerosols, these particles originate from diverse natural and anthropogenic reservoirs including soil, vegetation, water, waste materials, agricultural activities and human-associated environments. Once aerosolized, microorganisms may remain suspended in the atmosphere, associate with airborne particulate matter and undergo atmospheric transport before deposition (16–18). Biological particles therefore form part of the broader environmental exposure mixture encountered through inhalation, although they remain substantially less characterized than conventional chemical air pollutants.

Although studies have investigated microbial diversity and bioaerosol generation within formal landfill, composting, and waste treatment environments (5, 6, 11), direct field evidence from informal municipal waste-burning environments remains limited, particularly in African peri-urban communities.

This gap is relevant from an environmental-health perspective because informal burning often occurs near residential dwellings, schools, healthcare facilities, transport corridors and informal economic activities. Individuals in affected communities therefore encounter environmental pollution as a mixture rather than as isolated contaminants. Smoke, particulate matter, gaseous combustion products, resuspended dust and biological material may occur simultaneously. Characterizing the different components of this environmental mixture is an important prerequisite for subsequently understanding cumulative exposure and potential public-health relevance.

The issue also has an environmental-justice dimension. Informal waste burning frequently occurs in communities experiencing inadequate waste services, illegal dumping and broader environmental infrastructure deficits. Populations with limited ability to avoid or control these sources may consequently experience recurrent and overlapping environmental exposures. Expanding understanding of the biological component of the surrounding air may therefore contribute to more complete characterization of environmental burdens in under-served communities.

The need to address this knowledge gap is particularly relevant within South Africa, where informal waste burning remains a common consequence of inadequate waste management services, illegal dumping and rapid urbanization within many peri-urban and under-serviced communities (7, 8). These activities frequently occur in close proximity to residential areas, schools, healthcare facilities and informal economic activities, creating repeated opportunities for community exposure to combustion-derived air pollution. Although South Africa has established comprehensive legislative and policy frameworks for managing ambient air quality through the National Environmental Management: Air Quality Act (Act No. 39 of 2004) and the National Framework for Air Quality Management, these frameworks remain centered on chemical pollutants (3). Biological components of atmospheric pollution are rarely considered within routine air quality monitoring, environmental exposure assessment or regulatory practice.

Accordingly, this study investigated culturable airborne bacteria associated with active informal waste-burning environments in Hammanskraal, a peri-urban area within the City of Tshwane Metropolitan Municipality, South Africa. Specifically, the study sought to (i) quantify culturable airborne bacterial concentrations in active informal waste-burning environments relative to a non-burning comparison site, (ii) examine spatial variability in culturable bacterial concentrations across approximate distances from active combustion, and (iii) characterize representative culturable bacterial taxa recovered from the sampled environments. The study was designed as an exploratory environmental investigation rather than a quantitative health-risk or source-apportionment assessment. We hypothesized that active waste-burning environments would exhibit higher culturable bacterial concentrations than the comparison environment, while recognizing that plume behavior, combustion intensity, environmental disturbance and meteorological conditions could modify spatial patterns. By addressing an under-characterized biological dimension of these environments, the study provides baseline evidence for future integrated investigations of informal waste burning, environmental exposure and public health.

## 2 Materials and methods

### 2.1 Study area

The study was conducted in the broader Hammanskraal area within the City of Tshwane Metropolitan Municipality, Gauteng Province, South Africa. Hammanskraal comprises several interconnected peri-urban settlements, including Tema, Babelegi, Kudube and surrounding communities, and is characterized by rapid population growth, municipal service delivery challenges and persistent informal waste disposal and burning activities (7, 19).

Sampling was undertaken at three locations where active informal waste burning was observed and at one non-burning comparison site. The active sites represented recurrent informal waste-disposal and combustion environments identified within the study area (Figure 1). The comparison site was selected based on the absence of active waste burning or visible evidence of recent combustion during site assessment and sampling.

**Figure 1:**
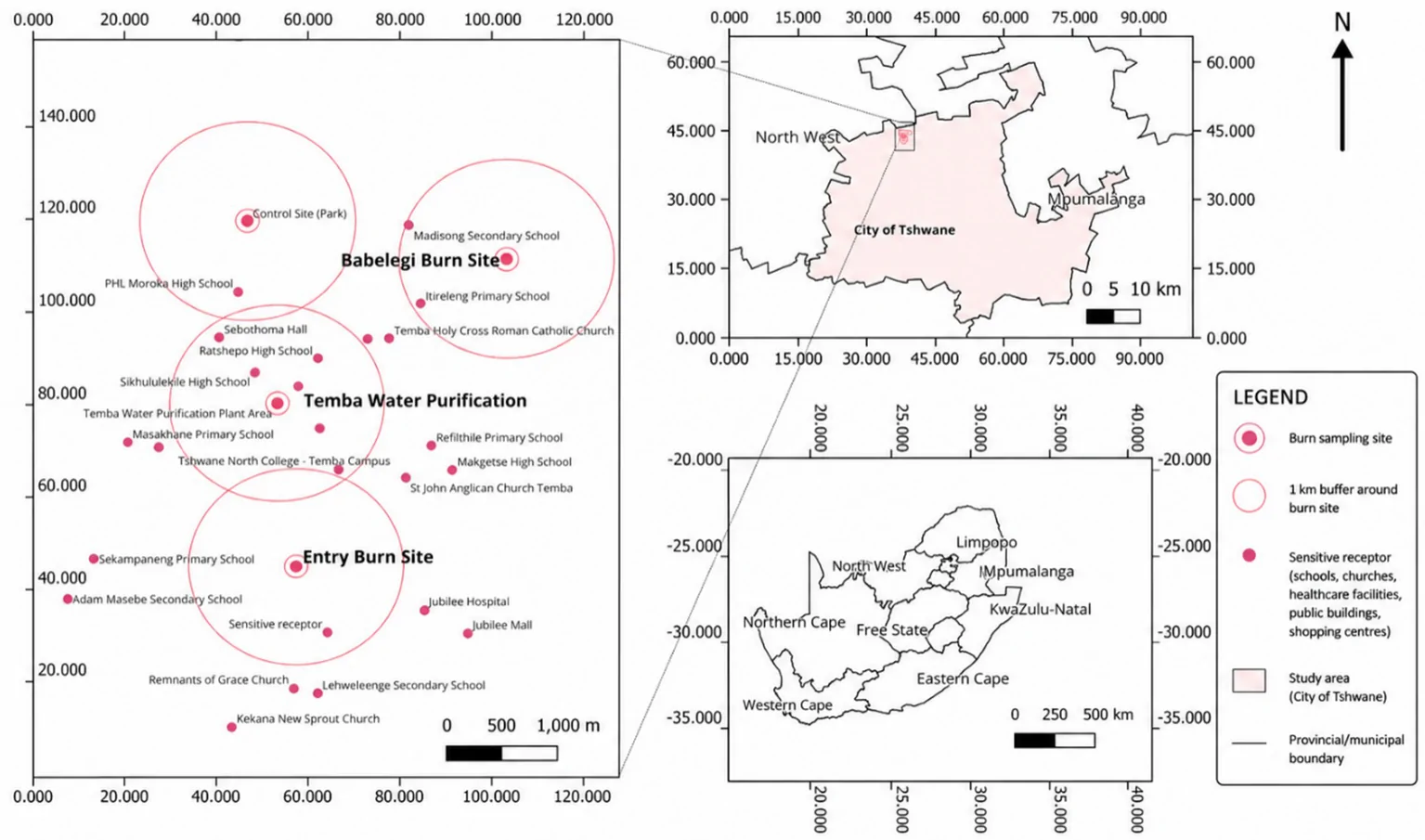
Informal waste-burning sampling sites, and non-burning comparison site within the Hammanskraal area. 1 km buffer zones illustrate the near-field community context surrounding each sampling location.

Because no combustion source was present at the comparison site, the nominal sampling positions (25, 50 and 100 m) represented approximately equivalent spatial intervals along the sampling transect rather than distances from a burn source. Background or comparison locations are commonly incorporated into environmental bioaerosol studies to characterize local ambient microbial conditions and provide a reference against which source-associated concentrations can be interpreted (20, 21). Accordingly, the equivalent spatial intervals employed at the comparison site in the present study were intended to maintain comparable sampling effort and capture local ambient variability rather than evaluate distance-dependent dispersion.

Figure 1 illustrates the spatial distribution of the three informal waste-burning sampling sites and comparison location. The sampled environments were located within a broader peri-urban landscape characterized by residential, institutional, transport and informal economic land uses. Educational institutions, healthcare facilities, residential settlements and transport corridors were located within the broader near-field context of the identified burning locations.

### 2.2 Study design and environmental exposure framework

A cross-sectional, event-based field sampling design was employed to characterize culturable airborne bacteria occurring during active informal waste-burning conditions. Cross-sectional, source-focused and activity-based sampling strategies have been used in outdoor bioaerosol investigations to characterize spatially and temporally variable microbial exposures associated with waste handling and other intermittent emission-generating activities (21–23). The investigation was designed as an exploratory environmental exposure study rather than an assessment of personal inhalation dose. Air sampling within the approximate human breathing zone and across nominal spatial intervals around active combustion was intended to characterize environmental conditions potentially relevant to inhalation exposure. The study did not include personal monitoring, epidemiological endpoints, pathogenicity testing or quantitative microbial risk assessment. Sampling locations were purposively selected according to the visible occurrence of recurrent informal waste-burning activity and field accessibility.

Field sampling was conducted on 9 February 2026 under warm, dry summer conditions. Sampling commenced at approximately 09:46 and concluded at approximately 14:00, with all active-burning and comparison locations sampled during a single field campaign. Because informal waste burning occurred unpredictably and varied in intensity throughout the day, the sequence of site visits was determined pragmatically according to active combustion conditions rather than predetermined site numbering. The sampling sequence was the comparison site, Site 2, Site 3 and Site 1. Site numbers therefore represent fixed location identifiers rather than sampling chronology.

Conducting sampling within a single campaign reduced broad day-to-day meteorological variability; however, short-term changes in combustion intensity, plume behavior, wind direction and atmospheric mixing may have contributed to between-site differences. Structured field observations included ambient temperature, relative humidity, wind speed and direction, visible smoke characteristics, combustion intensity, odor, waste materials and anthropogenic activities such as dumping, waste picking, pedestrian movement and traffic.

### 2.3 Spatial sampling strategy

Sampling positions were established at measured distances of 25, 50 and 100 m from the active waste-burning source using a mechanical distance-measuring wheel (WEBCO, 1000 mm wheel). Distances were measured along the sampling transect from the identified combustion source to each sampling position. Because the sampling sites were informal and spatially irregular, measurements followed the accessible field transect from the combustion area to each sampling position. Terrain, physical obstructions, active dumping and field-safety considerations influenced the direction of the sampling transect at some sites. The reported 25, 50 and 100 m values therefore represent measured field distances along the sampling transect, rather than geospatially derived radial distances from a fixed source coordinate. Distance-gradient sampling approaches have previously been used to evaluate spatial dispersion and attenuation patterns of airborne microorganisms and particulate-associated emissions in outdoor environments (16). The selected distance intervals were intended to provide a near-field spatial profile of culturable airborne bacterial concentrations under active waste-burning conditions. Where feasible, sampling positions were selected with consideration of visible smoke-plume movement. However, plume direction varied during sampling and could not be standardized experimentally.

At Site 2, a substantial change in visible plume direction occurred during the sampling period. Two separate air samples were consequently collected at the 50 m position under different plume-interaction conditions. These observations are referred to as 50a and 50b and were treated as separate environmental measurements rather than technical replicates.

### 2.4 Waste and environmental characterization

Waste typology at each burn site was documented using structured field observations. Observed materials were categorized into broad waste groups, including organic and food waste, vegetation, plastics and other polymer-based materials, paper, textiles, sanitary waste, tires, electronic waste, construction debris and mixed household refuse (Figure 2). These broad categories were used to characterize the environmental conditions present during sampling and to provide ecological context for interpretation of potential microbial reservoirs. Organic waste, vegetation, soil-contaminated material and sanitary waste are recognized as potential reservoirs of microorganisms in waste-associated environments (6, 11, 24). The categorization was not intended to attribute individual bacterial taxa to specific waste fractions. Rather, it provided a structured description of the heterogeneous materials occurring within each waste-burning environment.

**Figure 2:**
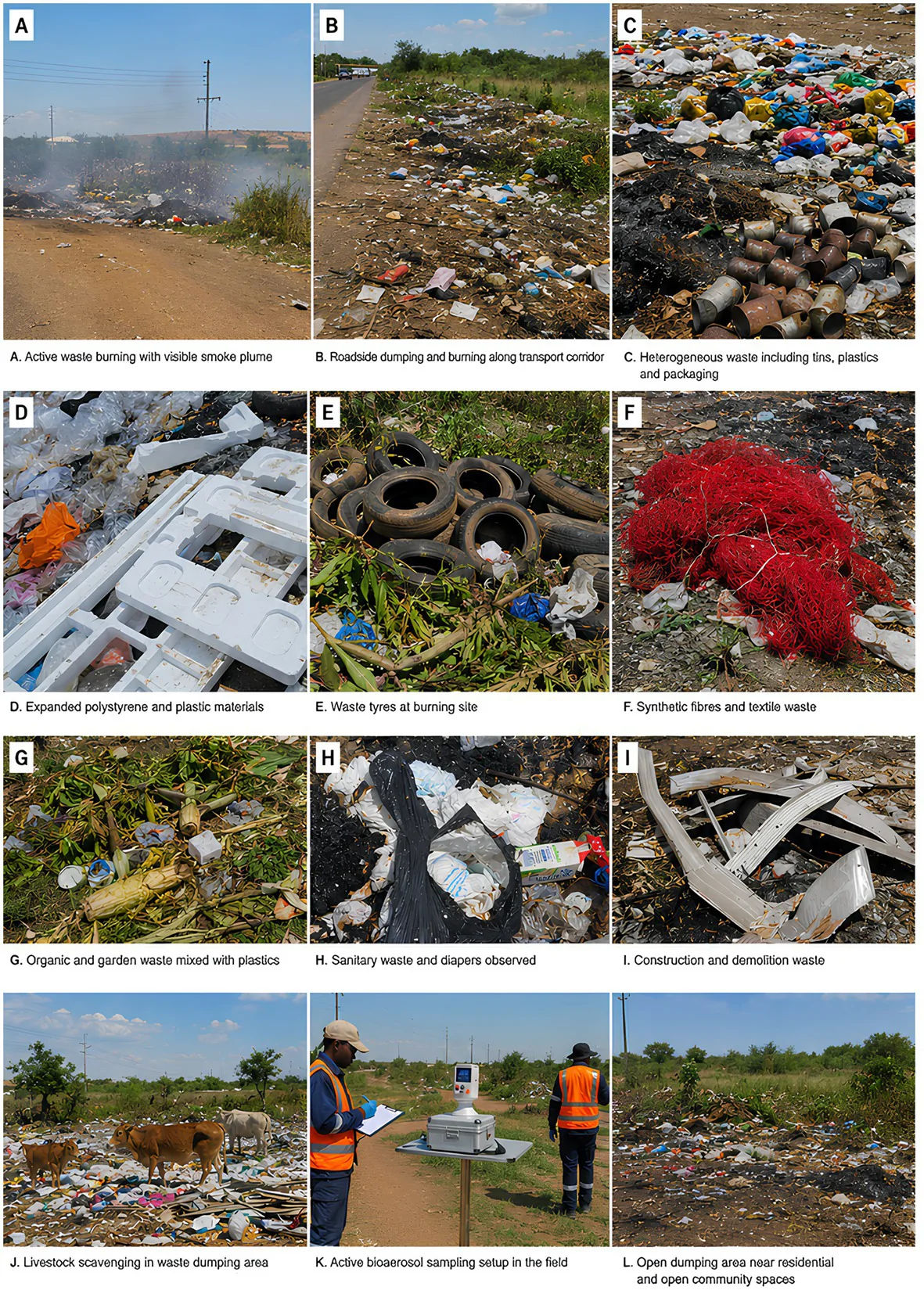
Representative field conditions across informal waste-burning environments, illustrating active combustion and smoke **(A)**, roadside dumping **(B)**, heterogeneous mixed waste **(C–I)**, animal activity within dumped waste **(J)**, active bioaerosol sampling **(K)**, and the surrounding open dumping environment **(L)**.

In addition to waste composition, field observations included combustion intensity, visible smoke characteristics, plume behavior, odor intensity, ambient temperature, relative humidity, wind speed and predominant wind direction. Anthropogenic activities including waste dumping, waste sorting, informal waste picking, pedestrian movement and vehicular activity were also documented where observed.

### 2.5 Bioaerosol sampling

Active bioaerosol sampling was undertaken using a Sampl’Air Pro air sampler (AES Chemunex, France), operated at a calibrated airflow rate of 100 L/min. During sampling, the instrument was positioned at an estimated height of approximately 1.5 m above ground level, broadly representing the human breathing zone. Similar sampler positioning has been used in previous outdoor bioaerosol investigations to characterize airborne microbial concentrations at heights relevant to human inhalation exposure (5).

Air samples were impacted directly onto sterile Tryptic Soy Agar (TSA) plates (Oxoid, UK), a general-purpose culture medium widely used for the recovery of heterotrophic airborne bacteria in environmental bioaerosol studies (10). Each sampling event was conducted for 10 min at 100 L/min, corresponding to a total aspirated air volume of approximately 1,000 L (1 m^3^) per sample. One active air sample was collected at each of the 25, 50 and 100 m sampling positions at each study site, except at Site 2, where an additional sample was obtained at 50 m following the substantial change in smoke-plume direction described in Section 2.3. No replicate active air samples were collected at individual site-distance positions; consequently, within-position sampling variability could not be estimated. Following sample collection, agar plates were immediately sealed, transported to the laboratory under controlled conditions and processed within 24 h of sampling to minimize viability loss and changes in microbial composition.

### 2.6 Culture-based enumeration and bacterial identification

Collected TSA plates were incubated aerobically at 37 °C for 18–24 h. Following incubation, visible bacterial colonies were enumerated, and culturable airborne bacterial concentrations were expressed as colony-forming units per cubic meter of sampled air (CFU/m^3^), based on the known sampled air volume. Where colony numbers exceeded the analytical upper quantification limit of the laboratory method, results were reported as >1,259 CFU/m^3^ in accordance with the laboratory reporting procedure. Culture-based enumeration remains widely used for the assessment of viable culturable airborne bacteria in environmental and waste-associated bioaerosol studies (4, 11, 22, 25). However, this approach recovers only the fraction of airborne microorganisms capable of remaining viable and growing under the selected culture and incubation conditions.

Following incubation and colony enumeration, a subset of colonies representing the range of culturable bacterial growth observed across the sampled plates was selected for further characterization. Selected colonies were subcultured onto Müller-Hinton Agar (Oxoid, UK) to obtain pure isolates for downstream identification. The resulting isolate set was used for descriptive characterization of recoverable culturable bacteria and was not intended to provide a systematic or quantitative representation of the total airborne bacterial community. Purified isolates were characterized using the Vitek Compact 2 automated microbial identification system (bioMérieux, France), using appropriate Gram-positive and Gram-negative identification cards in accordance with the manufacturer’s procedures. Isolates were identified to genus or species level where supported by the system outputs and corresponding biochemical profiles. Where definitive taxonomic identification was not achieved, isolates were conservatively retained within broader phenotypic categories, including Gram-positive bacilli. The frequencies reported for identified organisms therefore represent the frequency of representative cultured isolates selected for identification and should not be interpreted as the relative abundance, prevalence or complete diversity of the airborne bacterial community. This distinction is important because conventional culture-based approaches recover only a subset of environmental microbial diversity (4, 25, 26).

### 2.7 Quality assurance and control

Quality-assurance and contamination-control measures were implemented throughout field sampling, sample transport, laboratory processing and bacterial identification. The Sampl’Air Pro sampler was operated at a calibrated flow rate of 100 L/min and was calibrated before field deployment according to manufacturer specifications. Standardized sampling durations, airflow settings, incubation conditions and laboratory procedures were maintained throughout the field campaign to improve methodological consistency (10).

Before each active sampling event, the sampler head was disinfected with 70% ethanol and allowed to air dry before placement of the sterile agar plate. Non-sterile powder-free nitrile gloves were worn during sample handling, and agar plates were opened immediately before sampling to minimize unnecessary environmental exposure.

Field handling blanks were included at each sampling site to assess potential contamination associated with field handling, transport and laboratory processing. Blank TSA plates were exposed to ambient conditions for approximately 1 min without active air aspiration at the 50 m sampling position and were processed alongside active samples using the same transport and incubation procedures. Although the analytical laboratory reported blank observations using CFU/m^3^ notation, these results were interpreted qualitatively because no defined volume of air had been actively aspirated through the blank plates. Blank results were therefore used only to assess evidence of contamination and were not treated as measurements of ambient airborne bacterial concentration.

### 2.8 Statistical analysis

Descriptive statistical analyses were used to summarize culturable airborne bacterial concentrations across sampling sites and measured distance intervals (Figure 3). Concentration ranges and site-specific patterns were examined together with field observations to characterize spatial and environmental variability. Bacterial identification data were summarized using frequency counts, percentage occurrence among representative cultured isolates and site-occurrence patterns. Exploratory Spearman rank correlation and simple linear regression were used to examine the relationship between measured sampling distance and culturable airborne bacterial concentration at active waste-burning sites.

**Figure 3:**
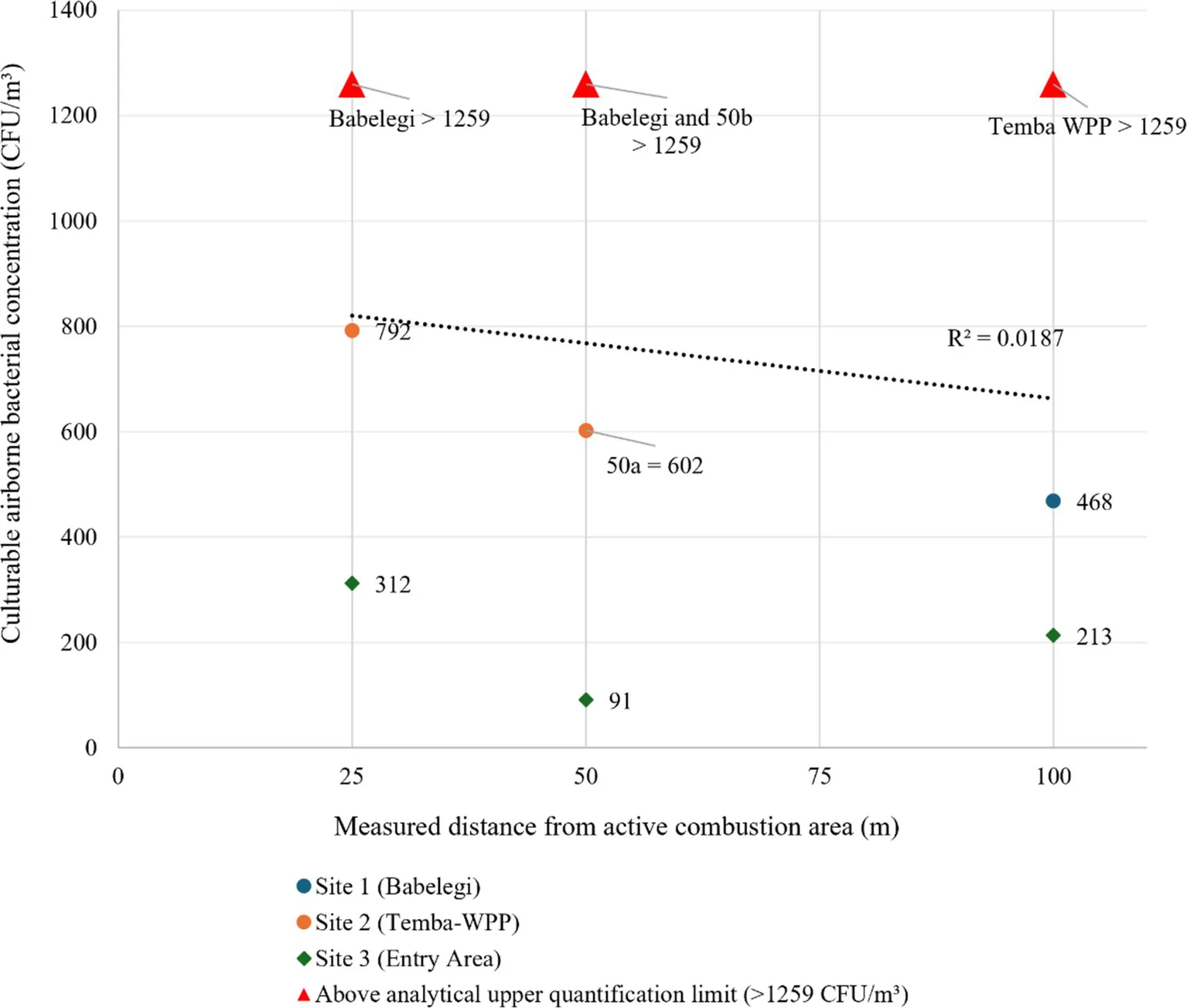
Exploratory relationship between measured sampling distance and culturable airborne bacterial concentration at active informal waste-burning sites. Distances of 25, 50 and 100 m were measured in the field using a measuring wheel. The dotted line represents an exploratory linear regression fitted to all active-burning observations (R^2^ = 0.02). Values reported above the analytical upper quantification limit (>1,259 CFU/m^3^) were plotted at 1,260 CFU/m^3^ solely for graphical presentation and exploratory statistical analysis. The fitted relationship should not be interpreted as a validated atmospheric dispersion model.

For results reported above the analytical upper quantification limit (>1,259 CFU/m^3^), a value of 1,260 CFU/m^3^ was assigned solely to permit graphical presentation and exploratory statistical analysis. This substituted value does not represent the true measured concentration and likely underestimates the magnitude of these right-censored observations. Because the study comprised a limited number of observations with restricted replication within individual site-distance combinations, inferential statistical analyses were considered exploratory and hypothesis-generating rather than confirmatory.

### 2.9 Ethical considerations

Ethical approval for the broader research project was granted by the University of Johannesburg Research Ethics Committee (Approval No. 2025-07-10/Kwakwa_Rampedi). Formal permission to undertake the study within the City of Tshwane Metropolitan Municipality was obtained from the City of Tshwane on 1 October 2025, prior to commencement of fieldwork. The present investigation involved environmental sampling only and did not include human participants, personal information or identifiable human biological material.

## 3 Results

### 3.1 Environmental and combustion characteristics of sampling sites

Field observations were recorded systematically during each sampling event to characterize the environmental conditions under which air sampling was conducted. These observations provide contextual information regarding combustion conditions, waste characteristics, plume behavior and site-specific factors potentially relevant to the observed airborne bacterial concentrations. A comparative summary is provided in Table 1, while representative field photographs are presented in Figure 2.

**Table 1:** Comparative environmental characteristics, combustion conditions and observed culturable airborne bacterial concentrations across active informal waste-burning sites and the non-burning comparison site.

Site	Burning intensity	Predominant waste materials	Key contextual feature	Temperature (°C)	Relative humidity (%)	Wind Speed (km/h)	Predominant wind direction	Smoke characteristics	Visibility	Odor intensity	Local setting	Culturable bacterial concentration range (CFU/m^3^ range)
Site 1: Babelegi	Moderate – high	Mixed household waste (plastics, paper, tires, vegetation, sanitary waste, hair fibers, polystyrene, construction debris)	Active waste dumping and waste picking adjacent to busy roadway	32–33	29–50	6–9	W–NW	White smoke; moderate to dense plume	Clear	Moderate–Strong	Gravel walkway approximately 1 m from tar road	468 – >1,259
Site 2: Temba Water Purification Plant (Temba-WPP)	Very high	Mixed household waste (organic waste, plastics, sanitary waste, grass, tires, aerosols, tins)	Highest combustion intensity; adjacent to Temba-WPP; plume shifts observed	31–32	31–33	3–5	N–NE	Dense grey smoke; persistent plume	Clear	Moderate–Strong	Gravel road and roadside sampling areas	602 – >1,259
Site 3: Entry Area	Moderate	Mixed household waste (plastics, tires, rubble, ceramics, fabrics, vegetation)	Multiple dispersed burn areas with intermittent plume interception	31–32	30–31	~5	NW	White smoke; intermittent plume	Clear	Mild–Strong	Gravel road and open land	91–312
Site 4: Comparison Site	None	Minor scattered litter (paper, plastics, rubble)	No active burning or waste disturbance observed	~30	~36	~4	NE	No visible smoke	Clear	Minimal/None observed	Gravel pathway within open vegetated area	35–260

#### 3.1.1 Site 1 (Babelegi) observations

Site 1 was characterized by active informal waste burning adjacent to a gravel access road located approximately 1 m from Mosimegi Street, a tarred roadway with frequent vehicle movement. Burning occurred concurrently with active waste dumping and informal waste picking, resulting in repeated disturbance of waste materials during the sampling period. The waste observed comprised a heterogeneous mixture of household refuse—including plastics, paper and polystyrene; tires and electronic waste; sanitary and textile waste—including diapers, shoes, blankets and household furnishings; vegetation and tree branches; and construction debris including ceiling boards and ceramic materials.

Sampling was conducted under warm, dry conditions with light winds and clear atmospheric conditions (Table 1). White smoke ranged from moderate to dense, and smoke-related odor was moderate to strong. Culturable airborne bacterial concentrations ranged from 468 CFU/m^3^ to above the analytical upper quantification limit (>1,259 CFU/m^3^) (see Table 2).

**Table 2:** Culturable airborne bacterial concentrations (CFU/m^3^) across sampling sites.

Site	Distance (m)	CFU/m^3^
Site 1 (Babelegi)	25	>1,259
Site 1	50	>1,259
Site 1	100	468
Site 2 (Temba-WPP Area)	25	792
Site 2	50a	602
Site 2	50b	>1,259
Site 2	100	>1,259
Site 3 (Entry Area)	25	312
Site 3	50	91
Site 3	100	213
Comparison site	25	35
Comparison site	50	189
Comparison site	100	260

Table 2 uses a heatmap-like colour gradient visual conditional formatting reflecting increasing CFU/m³ values only and do not represent regulatory, health-risk or exposure categories.

#### 3.1.2 Site 2 (Temba water purification plant) observations

Site 2 was located across the road from the Temba Water Purification Plant (Temba-WPP) along Makapanstad Road, and exhibited the greatest combustion intensity observed during the sampling campaign. This site was characterized by sustained open flames, dense grey smoke plumes and frequent shifts in plume direction throughout sampling.

Waste materials included mixed organic refuse, plastics, sanitary waste, vegetation, tires, tins and aerosol containers. Two localized explosive events occurred during sampling (approximately 10:58 and 11:06), in an area containing aerosol containers and other mixed waste. A substantial shift in smoke-plume direction occurred during sampling at the 50 m position. A second air sample was consequently collected at the same measured distance under a different plume-interaction condition.

#### 3.1.3 Site 3 (entry area) observations

Site 3 comprised several smaller active burning areas adjacent to Makapanstad Road and Harry Gwala Avenue. Observed waste included plastics, tires, paper, cans, polystyrene, vegetation, fabrics, rubble and other mixed municipal refuse. Smoke production was generally less persistent than at Site 2, although intersecting plumes from multiple smaller burns were observed intermittently. Culturable airborne bacterial concentrations ranged from 91 to 312 CFU/m^3^. Sampling was conducted under warm conditions with light north-westerly winds and generally clear weather.

#### 3.1.4 Comparison site observations

No active waste burning or visible evidence of recent combustion was observed at the comparison site during sampling. The site comprised open ground and mixed vegetation, with substantially less anthropogenic disturbance than the active-burning locations. No visible smoke or combustion-associated odor was recorded. Culturable airborne bacterial concentrations ranged from 35 to 260 CFU/m^3^.

#### 3.1.5 Cross-site environmental context

Meteorological and environmental conditions recorded during sampling are summarized in Table 1, while representative field photographs are presented in Figure 2. Across all study sites, temperatures remained relatively warm (30–33 °C) under generally clear conditions, with light wind speeds ranging from approximately 3–9 km/h. Despite these broadly similar meteorological conditions, differences were observed in combustion intensity, smoke-plume behavior, waste composition, proximity to road traffic, odor intensity and levels of anthropogenic disturbance.

Active waste dumping, waste sorting, informal waste picking, pedestrian movement, road traffic and dust resuspension were documented at several burn sites throughout the sampling campaign. Smoke-plume behavior also varied considerably among sites, with visible changes in plume direction occurring in response to local wind conditions. Intermittent plume interception at several sampling locations further highlighted the dynamic nature of environmental conditions within active waste-burning environments.

Collectively, these observations provide important environmental context for interpreting the variability in culturable airborne bacterial concentrations reported in the subsequent Results section. They indicate that the sampled waste-burning environments were characterized by multiple concurrent physical, atmospheric and anthropogenic processes that may plausibly have influenced short-term bioaerosol occurrence. These factors were therefore considered as contextual variables rather than as independently quantified determinants of bacterial concentration.

### 3.2 Culturable airborne bacterial concentrations

Culturable airborne bacteria were detected across all sampled environments. Distances at active-burning sites were measured in the field using a measuring wheel from the active combustion area along the accessible sampling transect. At the comparison site, 25, 50 and 100 m represent corresponding measured positions along the sampling transect because no combustion source was present. Site 2 observations 50a and 50b were collected at the same measured distance under different plume-interaction conditions. Concentrations ranged from 35 CFU/m^3^ at the comparison site to values exceeding the analytical upper quantification limit (>1,259 CFU/m^3^) at multiple active-burning sampling positions. At Site 1, concentrations exceeded >1,259 CFU/m^3^ at 25 and 50 m and decreased to 468 CFU/m^3^ at 100 m. At Site 2, concentrations were 792 CFU/m^3^ at 25 m, 602 and >1,259 CFU/m^3^ for the two observations collected at 50 m under different plume-interaction conditions, and >1,259 CFU/m^3^ at 100 m. Site 3 displayed lower concentrations of 312, 91 and 213 CFU/m^3^ at 25, 50 and 100 m, respectively. At the comparison site, concentrations recorded at the corresponding transect positions were 35, 189 and 260 CFU/m^3^.

At Site 2, two sampling measurements were obtained at approximately 50 m from the combustion source due to a substantial shift in visible plume direction during active burning. The second measurement therefore represented sampling under an alternate plume pathway rather than a technical replicate. This adjustment was undertaken to capture changing plume interaction conditions associated with dynamic combustion and meteorological behavior during the sampling period. Field handling blanks yielded 0–4 colonies, indicating minimal contamination associated with field handling and laboratory processing.

### 3.3 Active-burning and comparison environments

Culturable airborne bacterial concentrations at the non-burning comparison site ranged from 35 to 260 CFU/m^3^, whereas concentrations across the three active-burning sites ranged from 91 CFU/m^3^ to >1,259 CFU/m^3^. The highest concentrations occurred at Sites 1 and 2, where four observations exceeded the analytical upper quantification limit. Site 3 exhibited lower concentrations ranging from 91 to 312 CFU/m^3^ and overlapped with concentrations measured at the comparison site. Thus, although the highest culturable bacterial concentrations occurred within active-burning environments, substantial heterogeneity was observed both among and within sampling sites.

### 3.4 Spatial variability in culturable airborne bacterial concentrations

Spatial variability in culturable airborne bacterial concentrations was observed across the three active waste-burning sites. Site 1 exhibited the clearest decreasing pattern, with concentrations >1,259 CFU/m^3^ at measured distances of 25 and 50 m and 468 CFU/m^3^ at 100 m. In contrast, Site 2 showed no monotonic decline with increasing distance. Concentrations exceeded the analytical upper quantification limit at one of the two 50 m observations and at 100 m. The two samples collected at the same measured 50 m distance yielded concentrations of 602 and >1,259 CFU/m^3^ following a visible change in smoke-plume direction. Site 3 similarly displayed no monotonic distance pattern, with concentrations of 312, 91 and 213 CFU/m^3^ at 25, 50 and 100 m, respectively.

Exploratory statistical analysis indicated a weak negative association between measured sampling distance and culturable airborne bacterial concentration (Spearman’s ρ = −0.14, *p* = 0.701). Simple linear regression explained approximately 2% of the observed variation (R^2^ = 0.02).

### 3.5 Quality control findings

No colony growth was observed on field handling blanks collected at Site 1, Site 3 or the comparison site. Site 2 field handling blanks yielded 0–4 colonies. Because blank plates were exposed to ambient conditions without active volumetric air aspiration, blank results were interpreted qualitatively rather than as quantitative airborne bacterial concentrations. The colony numbers observed on Site 2 blanks were substantially lower than those recorded on corresponding active samples. Overall, field handling blank results provided no evidence of substantial contamination arising from sample handling, transport or laboratory processing.

### 3.6 Characterization of selected culturable bacterial isolates

Culture-based identification of selected isolates recovered several bacterial taxa and unresolved phenotypic groupings across active-burning and comparison environments. A total of 39 cultured bacterial isolates were identified across all samples (Table 3). Percentages represent frequencies within the set of 39 cultured isolates selected for laboratory identification and should not be interpreted as relative abundance or prevalence within the total airborne bacterial community.

**Table 3:** Frequency distribution and site occurrence of identified airborne bacterial isolates and unresolved groupings.

Taxon/classification	Number of representative isolates (*n*)	Frequency among representative isolates (%)	Site 1 (Babelegi)	Site 2 (Temba-WPP)	Site 3 (entry)	Site 4 (comparison site)
*Gram-positive bacilli*	18	46.2	Yes	Yes	Yes	Yes
*Sphingomonas paucimobilis*	9	23.1	Yes	Yes	Yes	Yes
*Pantoea* spp.	4	10.3	No	Yes	Yes	Yes
*Rothia kristinae*	2	5.1	No	Yes	No	Yes
*Acinetobacter radioresistens*	2	5.1	No	No	Yes	No
*Kocuria rosea*	1	2.6	No	No	Yes	No
*Micrococcus luteus*	1	2.6	No	Yes	No	No
*Pseudomonas putida*	1	2.6	No	No	No	Yes
*Staphylococcus gallinarum*	1	2.6	No	No	No	Yes

Green shading indicates that the taxon/classification was represented among selected isolates from the site; red shading indicates that it was not represented among the selected isolates.

Among the selected isolates, Gram-positive bacilli represented the most frequently recorded phenotypic grouping, comprising 18 of 39 isolates (46.2%). *Sphingomonas paucimobilis* accounted for nine isolates (23.1%), followed by Pantoea spp. with four isolates (10.3%). Rothia kristinae and *Acinetobacter radioresistens* were each represented by two isolates (5.1%). *Kocuria rosea, Micrococcus luteus, Pseudomonas putida* and *Staphylococcus gallinarum* were each represented by one isolate (2.6%).

Gram-positive bacilli and *Sphingomonas paucimobilis* were represented among selected isolates from all four sampled environments. *Pantoea* spp. was represented at Site 2, Site 3 and the comparison site, while *Rothia kristinae* occurred among isolates from Site 2 and the comparison site. *Acinetobacter radioresistens* and *Kocuria rosea* were represented only among Site 3 isolates, whereas *Pseudomonas putida* and *Staphylococcus gallinarum* occurred only among isolates from the comparison site.

## 4 Discussion

### 4.1 Active informal waste-burning environments and culturable airborne bacterial occurrence

To our knowledge, this study provides the first field-based evidence from South Africa characterizing culturable airborne bacterial concentrations in active informal waste-burning environments. Informal waste burning is well established as a source of particulate matter, trace gases and hazardous combustion products (9), yet its potential biological component has received considerably less attention. The present findings extend this understanding by demonstrating that viable culturable bacteria are also present in air sampled during active informal waste-burning conditions.

Culturable airborne bacterial concentrations were generally elevated at the active-burning sites relative to the non-burning comparison environment. Several observations at Sites 1 and 2 exceeded the analytical upper quantification limit (>1,259 CFU/m^3^), whereas concentrations at the comparison site ranged from 35 to 260 CFU/m^3^. This pattern was not uniform, however. Concentrations at Site 3 ranged from 91 to 312 CFU/m^3^ and overlapped with those measured at the comparison site, demonstrating substantial heterogeneity among active-burning environments. The findings therefore do not support a simple interpretation in which the presence of combustion consistently corresponds to elevated bacterial concentrations. Instead, they demonstrate substantial heterogeneity among waste-burning environments and suggest that bacterial occurrence reflects site-specific environmental conditions.

Bioaerosols are ubiquitous components of the atmosphere and may originate from soil, vegetation, water, organic matter and anthropogenic activities (4). Waste environments provide particularly diverse potential microbial reservoirs. Waste-management environments are recognized sources of bioaerosols, with microbial concentrations influenced by waste characteristics, operational activities and meteorological conditions (11). This is relevant to the present study because active dumping, waste sorting, informal waste picking, road traffic and dust resuspension were observed at several sampled burn sites. These activities provide plausible mechanisms through which microorganisms associated with waste and surrounding environmental reservoirs may become airborne.

Combustion-associated processes may provide an additional mechanism for microbial mobilization and atmospheric transport. Evidence from wildland-fire environments demonstrates that viable microorganisms can be aerosolized and transported in smoke (27, 28). More recently, Kobziar et al. (29) reported approximately four-fold higher microbial cell concentrations in smoke from high-intensity forest fires than in background air, with approximately 78% of smoke-associated microbial cells inferred to be viable. These studies concern wildland biomass combustion rather than mixed municipal waste burning, and their findings should therefore not be directly extrapolated to the present study. Nevertheless, they provide mechanistic evidence that viable microorganisms can persist within combustion-affected air masses and that fire-associated atmospheric processes can facilitate microbial aerosolization and atmospheric transport.

The present findings do not establish that combustion itself generated the bacteria detected. The sampled sites contained multiple potential microbial reservoirs, including soil, vegetation, decomposing organic material, sanitary waste and mixed household refuse, while these materials were simultaneously disturbed through dumping, sorting, waste picking, traffic and other activities. Combustion may nevertheless modify how microorganisms from these reservoirs enter and move through the atmosphere. Thermal convection, turbulent airflow and smoke-plume movement provide plausible mechanisms for particle entrainment and transport, while meteorological conditions influence subsequent dispersion and dilution.

Culturable bacterial concentrations measured during this study should therefore be interpreted as the product of a complex environmental setting rather than as direct measurements of a discrete microbiological emission from combustion. The principal contribution of the findings is consequently the identification of viable culturable bacteria as an under-characterized component of the airborne environment associated with active informal waste burning. This establishes an important basis for investigating how waste characteristics, combustion, physical disturbance and atmospheric processes interact to influence biological airborne exposure.

### 4.2 Spatial variability: measured distance, plume behavior and site conditions

The second objective was to examine spatial variability in culturable bacterial concentrations across measured distances from active combustion. No consistent decline in concentration with increasing distance was observed across the three active-burning sites. The Spearman correlation indicated only a weak negative association between distance and bacterial concentration (ρ = −0.14, *p* = 0.701), while the exploratory linear model explained approximately 2% of the observed variation (R^2^ = 0.02). Within the conditions represented by this dataset, measured distance from the active combustion area alone therefore provided little explanatory value for short-term variation in bacterial concentrations.

This finding should not be interpreted as evidence that distance is unimportant to atmospheric dispersion. Rather, geometric distance did not necessarily correspond to the degree of plume interaction experienced at a sampling position. Site 2 provides a particularly clear illustration. Two samples collected at the same measured distance of approximately 50 m yielded concentrations of 602 and >1,259 CFU/m^3^ following a visible change in plume direction. Concentrations above the analytical upper quantification limit were also observed at 100 m during sustained combustion. Conversely, Site 1 showed a more conventional spatial pattern, with concentrations >1,259 CFU/m^3^ at 25 and 50 m and a lower concentration of 468 CFU/m^3^ at 100 m, while Site 3 showed no monotonic pattern.

The measured 25, 50 and 100 m intervals represented field distances along accessible sampling transects rather than continuously modelled downwind distances relative to the changing plume. This distinction is important in uncontrolled outdoor environments, where wind direction, source intensity and plume trajectory can change over relatively short periods. A sampling point located farther from the physical combustion area may therefore experience greater plume interception than a geographically closer position under particular wind conditions.

Between-site differences further indicate that distance alone cannot adequately characterize these environments. Sites 1 and 2 contained the highest observed bacterial concentrations and were characterized by greater combustion intensity, denser or more persistent smoke and substantial waste disturbance. Site 3, by contrast, contained smaller and more dispersed burning areas and generally lower bacterial concentrations. These observations provide plausible environmental context but do not demonstrate that combustion intensity, plume behavior or disturbance independently determined bacterial concentrations because these factors were not quantitatively modelled.

The spatial findings therefore emphasize the need to distinguish source distance from effective atmospheric exposure. Future studies should combine measured distance with repeated sampling, continuous wind measurements, plume tracking and concurrent particulate measurements to determine how downwind position and changing atmospheric conditions influence bioaerosol concentrations around informal waste-burning sources.

### 4.3 Interpretation of the selected culturable bacterial isolates

The selected culturable bacterial isolates recovered during this study illustrate the ecological diversity of microorganisms recovered from the sampled informal waste-burning and comparison environments. Because bacterial identification was performed on a selected subset of cultured colonies rather than on the entire recoverable bacterial population, the findings should not be interpreted as a quantitative characterization of airborne bacterial community composition. Instead, they provide qualitative evidence of the types of viable culturable bacteria recovered across the sampled environments.

The identified isolates comprised environmentally widespread organisms and taxa also reported from human-, animal- and waste-associated environments, consistent with the observed heterogeneous nature of the sampled sites, where soil, decomposing organic matter, vegetation, sanitary waste, household refuse and human activities coexisted within disturbed environmental settings. The recovered isolate set is therefore compatible with contributions from multiple environmental reservoirs rather than with a distinct combustion-specific bacterial assemblage.

Gram-positive bacilli represented the largest phenotypic grouping among the 39 selected isolates, accounting for 18 isolates (46.2%). This proportion should not be interpreted as evidence that Gram-positive bacilli dominated the total airborne bacterial community because it is influenced by the culture conditions and colony-selection process. Gram-positive bacteria are frequently reported in atmospheric bioaerosols, and characteristics including robust cell-wall structures, environmental stress tolerance and, for some taxa, spore formation may favor persistence under airborne conditions (4, 30). However, the present dataset cannot establish whether environmental selection associated with combustion was responsible for the number of Gram-positive bacilli represented among the selected isolates. Moreover, because only culturable bacteria were assessed using culture-dependent methods, the recovered isolates represent only a subset of the broader microbial assemblage potentially present in the sampled air.

Among the taxa resolved to species or genus level, *Sphingomonas paucimobilis* was represented among selected isolates from all four sampling environments, including the non-burning comparison site. Members of the genus *Sphingomonas* are widely distributed across soil, water, vegetation and atmospheric environments (13). Its occurrence at both active-burning and comparison sites is therefore more consistent with a broadly distributed environmental organism than with a bacterium uniquely associated with informal waste burning. Furthermore, the present study cannot determine whether its frequency among the selected isolates reflects its actual abundance in the sampled air.

Similarly, *Pantoea* spp. were recovered among selected isolates from Site 2, Site 3 and the comparison site, but not among those identified from Site 1. Species within the genus *Pantoea* are commonly associated with plants, soil and organic substrates (31). Their occurrence is ecologically consistent with the vegetation and organic material observed across several sampling environments, although the study was not designed to attribute these isolates to particular waste fractions or environmental sources. The recovery of *Pantoea* spp. at the comparison site further indicates that their presence should not be interpreted as a microbial marker specific to waste-burning activity.

The remaining identified taxa further demonstrate the range of culturable organisms recovered from the sampled environments. *Micrococcus luteus* and *Kocuria rosea* have been reported from environmental surfaces, dust and human-associated environments (13, 32, 33), while *Pseudomonas putida* is widely associated with soil and water (34). *Acinetobacter radioresistens, Rothia kristinae* and *Staphylococcus gallinarum* were also identified among the selected isolates. Given the low number of isolates represented by these taxa and the absence of microbial source-tracking, their occurrence should be interpreted descriptively rather than as evidence of particular waste sources, exposure pathways or health hazards.

The occurrence of identified taxa across both active-burning and comparison environments is an important finding. It indicates that the culturable bacteria recovered during active waste burning were not composed exclusively of organisms absent from the surrounding ambient environment. However, because colony selection for Vitek identification did not constitute a quantitative community-sampling approach, the present study cannot determine whether informal waste burning altered bacterial community composition, taxonomic richness or relative abundance. Such comparisons would require standardized isolate-selection procedures or, preferably, culture-independent molecular characterization.

The occurrence of several taxa across both active-burning and comparison environments is itself informative. It indicates that the culturable organisms recovered during active burning were not composed exclusively of bacteria absent from the surrounding ambient environment. The present study therefore provides no evidence of a unique “waste-burning microbiome.” Determining whether informal waste burning alters airborne bacterial community composition, richness or relative abundance would require systematic community-level characterization using culture-independent approaches such as 16S rRNA amplicon sequencing or shotgun metagenomics, ideally combined with viability-sensitive and microbial source-tracking methods.

### 4.4 Opportunistic taxa and exposure relevance

Although the majority of bacteria recovered during the present study comprised environmentally associated taxa, several opportunistic microorganisms were also identified within both active waste-burning and comparison-site samples. Opportunistic bacteria are widely distributed in environmental and host-associated reservoirs and, by definition, typically cause infection when host defenses are impaired or other conditions favor pathogenicity (35). Detection in environmental air therefore does not, by itself, demonstrate pathogenicity, infection or public-health risk.

The environmental relevance of these findings therefore lies primarily in identifying an additional biological component within settings already affected by established combustion pollutants. Bioaerosol exposure in occupational and environmental settings has been associated with respiratory and inflammatory effects, although exposure-response relationships remain insufficiently characterized to establish broadly applicable health-based ambient limits (25, 36). Accordingly, the bacterial concentrations measured in the present study should not be interpreted as health-risk thresholds.

The identification results instead provide a basis for subsequent exposure-focused research. Future investigations incorporating personal or breathing-zone monitoring, molecular characterization, viability assessment and, where justified, quantitative microbial risk approaches would be required before the health significance of individual organisms or measured bacterial concentrations could be established.

### 4.5 Waste heterogeneity and potential microbial reservoirs

The heterogeneous composition of waste observed across the informal burning sites provides important context for interpreting the culturable airborne bacteria recovered during this study. Unlike engineered waste-management systems, where waste streams may undergo greater separation or controlled processing, informal waste-burning environments typically comprise highly variable mixtures of household refuse, organic waste, vegetation, plastics, textiles, sanitary waste, tires, construction debris and other discarded materials occupying the same physical space. These heterogeneous waste assemblages represent potential microbial reservoirs originating from multiple environmental and anthropogenic sources. Consequently, the culturable bacteria recovered from these environments may reflect contributions from multiple waste and environmental reservoirs rather than from any single waste category (Figure 2) (25).

From an ecological perspective, different waste fractions may provide distinct microbial reservoirs prior to combustion. Organic waste and decomposing food residues provide favorable conditions for microbial growth and decomposition, while vegetation and plant material commonly harbor plant-associated bacteria and fungi (22). Soil and soil-contaminated materials may introduce terrestrial microorganisms (31), whereas sanitary waste may provide additional human- or animal-associated microbial reservoirs. The co-occurrence of these materials within mixed waste environments therefore creates multiple potential sources from which microorganisms may subsequently become airborne (37). Plastics, textiles and other non-organic materials may additionally provide surfaces for microbial attachment and persistence, with associated microorganisms potentially becoming resuspended following physical disturbance.

These observations argue against conceptualizing informal waste-burning locations as simple point sources. The sites examined here were simultaneously dumping areas, waste-sorting environments, combustion zones, pedestrian spaces and, in some cases, roadside environments. Waste picking, dumping, vehicle movement, pedestrian activity and wind-driven resuspension repeatedly disturbed accumulated materials. Combustion added thermal uplift, turbulence and smoke-plume movement to these existing disturbance processes. The airborne bacteria recovered at any individual sampling position could therefore have reflected contributions from several reservoirs and mobilization mechanisms operating concurrently.

Figure 4 synthesizes this interpretation by proposing pathways through which heterogeneous waste reservoirs, physical disturbance, combustion-associated atmospheric processes and meteorological conditions may contribute to microbial aerosolization, transport and potential human contact. The framework intentionally distinguishes plausible mechanisms from demonstrated causal relationships. Waste composition and site activities were observed, and culturable bacteria were measured in the surrounding air; however, the study did not quantify the contribution of individual waste fractions or determine the specific source of recovered organisms.

**Figure 4:**
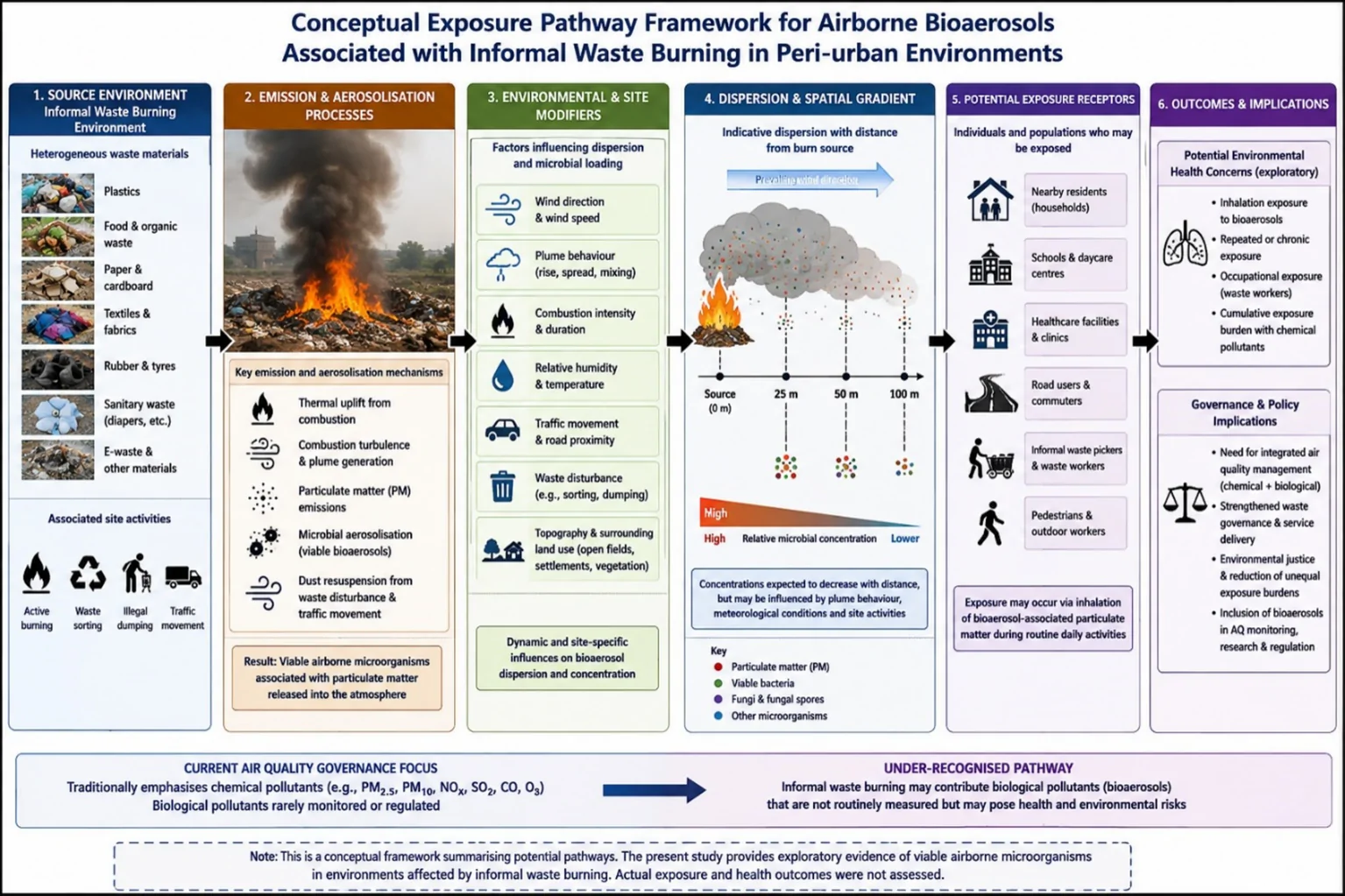
Conceptual exposure pathway framework for airborne bioaerosols associated with informal waste burning in peri-urban environments. The illustration was generated using OpenAI’s image-generation tool within ChatGPT and reviewed by the authors for scientific accuracy.

Waste typology should therefore be considered not merely as a description of what was burned but as a characteristic of the broader microbial source environment. Future source-material sampling of waste fractions, soil, vegetation and background air, combined with microbial source-tracking approaches, would enable the relative contributions of these reservoirs to airborne bacterial communities to be evaluated.

### 4.6 Interacting environmental factors influencing airborne bacterial occurrence

The findings of the present study indicate that culturable airborne bacterial concentrations within informal waste-burning environments are unlikely to be determined by any single environmental factor. Instead, the observed spatial variability is consistent with the potential influence of multiple physical, environmental and anthropogenic processes operating simultaneously. Waste composition, combustion intensity, meteorological conditions, plume behavior and ongoing site disturbance all varied between sampling locations and provide important context for the variability in bacterial concentrations observed during field sampling. Because these factors were not independently quantified as predictors, their individual contributions to bacterial occurrence cannot be determined from the present dataset. Consequently, informal waste-burning environments may be better understood as dynamic environmental systems in which multiple interacting processes potentially influence the occurrence and transport of airborne microorganisms.

Among the factors observed during field sampling, combustion intensity varied notably among sites and coincided with differences in culturable bacterial concentrations. Sites 1 and 2, which exhibited moderate-to-high or high combustion intensity and substantial smoke production, contained several of the highest bacterial concentrations, whereas Site 3 was characterized by smaller, more dispersed burns and lower concentrations. However, these observations do not establish combustion intensity as an independent determinant of bacterial loading. Active combustion generates thermal energy and buoyant smoke plumes that can influence the entrainment and atmospheric transport of particles and potentially associated biological material. Evidence from wildland-fire environments further indicates that viable microorganisms can occur within and be transported by combustion-affected air masses (27–29). These mechanisms provide a plausible basis for interpreting the field observations but were not directly measured in the present study.

Plume behavior provided particularly useful field context for interpreting short-term variability. Throughout the sampling campaign, visible smoke plumes changed direction in response to local wind conditions, altering whether individual sampling positions visibly intercepted the plume. The two samples collected at the measured 50 m position at Site 2 illustrate this variability: concentrations of 602 and >1,259 CFU/m^3^ were recorded at the same distance following a visible change in plume direction. These observations suggest that geometric distance from the active combustion area did not necessarily correspond to equivalent plume interaction during sampling. They should not, however, be interpreted as demonstrating that plume direction caused the difference in bacterial concentration, because plume exposure was observed qualitatively rather than quantitatively measured. Nevertheless, the observation highlights the limitations of interpreting short-term bioaerosol concentrations solely according to distance from an active combustion area.

Meteorological conditions provide an additional interpretive factor. As shown in Table 1, temperature, relative humidity, wind speed and predominant wind direction were recorded during field sampling and used to characterize the environmental conditions prevailing at each site. Although temperature and relative humidity were broadly similar across the sampling campaign, local differences in wind speed and predominant wind direction were observed. Wind speed and wind direction are established determinants of atmospheric transport and dispersion and may therefore have contributed to the observed variability in culturable airborne bacterial concentrations, particularly through their influence on plume trajectory, dilution and downwind transport (4, 15). The observed changes in smoke-plume direction during sampling, particularly at Site 2, further demonstrate that sampling positions were subject to temporally variable plume interaction. In the present study, meteorological variables were collected to characterize prevailing field conditions rather than to test meteorological determinants of bacterial concentration. The limited number of observations and absence of temporal replication did not support robust multivariable analysis. Meteorological measurements were therefore used descriptively and should not be interpreted as statistically evaluated predictors.

Physical disturbance provides another plausible pathway for microbial aerosolization and resuspension. Active waste dumping, waste sorting, pedestrian movement, waste picking and vehicular traffic were documented at several sampling locations. Waste-management studies have demonstrated that operational activities and physical disturbance can contribute to bioaerosol generation and resuspension (11, 21). Within informal waste-burning environments, these activities occurred concurrently with combustion and therefore could not be separated as independent contributors to the bacterial concentrations measured. The observed concentrations may consequently reflect contributions from both disturbance-related resuspension and combustion-associated atmospheric processes, although their relative importance remains unresolved.

Collectively, these observations reinforce the interpretation of informal waste-burning sites as dynamic environmental systems. The measured bacterial concentration at a particular point and time may reflect the interaction of available microbial reservoirs, physical disturbance, combustion behavior and atmospheric transport rather than any single determinant. Future studies should therefore employ greater temporal and spatial replication together with continuous meteorological monitoring, quantitative plume characterization and concurrent particulate measurements. Such designs would enable multivariable modelling of wind speed, wind direction, temperature, relative humidity, combustion characteristics and disturbance processes and allow their independent and interacting relationships with airborne bacterial concentrations to be evaluated.

### 4.7 Implications for bioaerosol exposure and health risk

The principal environmental health significance of the present study lies not in demonstrating disease causation, but in expanding current understanding of the airborne environmental conditions associated with informal waste burning. Existing research has predominantly focused on chemical pollutants generated during open waste combustion, including particulate matter, carbon monoxide, hydrocarbons, reactive trace gases, and other hazardous combustion products (8, 9, 38). The present findings add a biological dimension to this literature by demonstrating that viable culturable bacteria can also occur within active informal waste-burning environments.

This observation is relevant because real-world exposure occurs within environmental mixtures rather than as isolated contact with individual pollutants. Particulate matter itself can transport microorganisms, microbial fragments, endotoxins and other biologically active material (4, 39). The present study did not determine whether recovered bacteria were attached to combustion-derived particles or whether biological and chemical pollutant concentrations covaried. Nevertheless, their coexistence within active waste-burning settings supports consideration of bioaerosols in future integrated exposure assessments.

Potential exposure is particularly relevant where recurrent burning occurs near residential areas, schools, healthcare facilities, transport corridors and informal economic activities. Several such receptors occurred within the broader surroundings of the sampled locations. Residents and individuals whose livelihoods involve waste picking, sorting or recycling may consequently have repeated opportunities for contact with smoke, disturbed waste material and airborne microorganisms. However, environmental occurrence should not be conflated with personal exposure: the present study did not measure individual inhalation, exposure frequency, inhaled microbial dose or resulting health outcomes.

This distinction also constrains interpretation of the opportunistic taxa recovered. The study was not designed to assess pathogenicity, infectivity or quantitative microbial risk, and the presence of an opportunistic organism does not imply disease. Instead, the findings establish an earlier step in the exposure pathway: viable culturable bacteria were present in environmental air during active waste-burning conditions. Whether nearby individuals inhaled these organisms, in what quantities and with what biological consequences remains unknown.

The findings are also relevant to cumulative environmental exposure and environmental justice. Informal waste burning frequently occurs in settings affected by inadequate waste collection, illegal dumping and broader infrastructural constraints. South African research has documented the co-occurrence of pollution, inadequate waste services and other environmental burdens in disadvantaged informal-settlement contexts (40), while waste-related environmental inequalities are increasingly recognized internationally as an environmental-justice concern (41). Where recurrent burning overlaps with residential and livelihood activities, affected populations may experience multiple environmental burdens while having limited ability to avoid or control their sources.

The findings therefore do not justify microbial health thresholds or imply an immediate requirement for routine bioaerosol monitoring. Rather, they identify viable culturable bacteria as an under-characterized component that warrants inclusion in future integrated exposure research. Combining microbial characterization with chemical pollutant measurements, meteorology and personal-exposure assessment would enable the public-health relevance of this biological component to be evaluated more rigorously.

### 4.8 Implications for air-quality and waste management

The findings of the present study have important implications for the way informal waste burning is conceptualized within contemporary air quality management. South Africa’s National Environmental Management: Air Quality Act (Act No. 39 of 2004; NEM:AQA) establishes an effects-based framework for protecting health and the environment through air-quality management, monitoring and standard setting. In practice, national ambient air-quality standards and routine monitoring are centered on established criteria pollutants, including sulfur dioxide (SO₂), nitrogen dioxide (NO₂), ozone (O₃), carbon monoxide (CO), lead (Pb), particulate matter (PM₁₀ and PM₂.₅) and benzene (C₆H₆) (3). This emphasis is appropriate given the substantial evidence linking these pollutants to adverse health and environmental outcomes. However, the present findings suggest that informal waste-burning environments may contain an additional biological component that is not routinely characterized within conventional air-quality monitoring.

This distinction has practical implications. Routine inclusion of microbial measurements within municipal ambient air-quality networks would currently be premature because standardized monitoring approaches, health-based ambient thresholds and sufficiently characterized exposure-response relationships remain limited. The immediate management implication is therefore not the creation of microbial air-quality standards, but improved prevention of the environmental conditions giving rise to uncontrolled burning and more integrated investigation of affected locations.

The management implication is therefore primarily one of source prevention and integrated exposure characterization, rather than immediate expansion of routine regulatory monitoring. Open waste burning is already recognized as a significant source of particulate matter, gases and hazardous combustion products (8, 9, 38). Measures that improve waste collection, reduce illegal dumping and prevent uncontrolled burning are consequently justified on established air-quality and waste-management grounds. The present findings suggest that such interventions may also reduce opportunities for microbial mobilization associated with disturbed waste environments, although this potential co-benefit was not quantified in the present study. This reinforces the value of linking air-quality management with waste-service provision, land-use planning and community-level source prevention rather than treating informal waste burning solely as an atmospheric monitoring problem.

This shifts management emphasis upstream. Informal waste burning is not solely an atmospheric monitoring problem; it is also associated with waste-service provision, illegal dumping, land use and local environmental management. Where recurrent burning occurs near sensitive receptors, targeted hotspot investigations could combine conventional pollutant measurements with bioaerosol sampling and detailed site characterization. Such research would provide stronger evidence regarding whether biological and chemical components vary concurrently and whether particular site conditions warrant additional management attention.

Environmental justice strengthens the rationale for this integrated approach. Communities affected by recurrent informal waste burning may simultaneously experience inadequate waste services and multiple environmental pollution sources (40, 41). Source prevention therefore has potential benefits beyond compliance with individual ambient standards by addressing an upstream environmental condition contributing to overlapping exposures.

The principal management contribution of this study is thus conceptual rather than regulatory. Informal waste-burning environments should not be understood exclusively through the conventional chemical-pollutant lens. They may also contain viable airborne biological material arising from the interaction of heterogeneous waste reservoirs, disturbance and atmospheric processes. Recognizing this additional dimension can inform targeted research and more integrated waste, air-quality and exposure-management strategies without implying that existing air-quality legislation requires immediate amendment.

### 4.9 Study limitations and priorities for future research

The findings of the present study should be interpreted within the context of its exploratory, cross-sectional design. The investigation was intended to provide baseline evidence of culturable airborne bacterial occurrence within active informal waste-burning environments rather than to establish source attribution, long-term exposure patterns or causal relationships. Several methodological limitations therefore define the extent to which the findings can be generalized and, importantly, identify priorities for subsequent research.

First, the temporal and spatial replication was limited. Sampling was conducted during a single field campaign and captured conditions associated with individual active-burning events at three sites and one non-burning comparison location. Informal waste burning is inherently variable, with waste composition, combustion intensity, human activity and atmospheric conditions changing between burning events, days and seasons. The concentrations reported here should therefore be interpreted as event-specific observations rather than estimates of typical or long-term airborne bacterial concentrations within the study area. Future investigations should incorporate repeated sampling across multiple burning events, seasons and times of day, together with additional comparison locations, to establish the temporal reproducibility and wider spatial representativeness of the observed patterns.

Meteorological conditions represent an important consideration in interpreting the observed variability in culturable airborne bacterial concentrations. Wind speed and wind direction are well-established influences on the transport and dispersion of airborne particles and bioaerosols and are therefore relevant to the spatial variability of airborne bacterial concentrations (4, 30). Meteorological variables, including wind speed, wind direction, temperature and relative humidity, were recorded during field sampling to characterize the prevailing environmental conditions. However, quantifying the independent effects of meteorological variables on bacterial concentrations was not an objective of the present exploratory study, and the sampling design and number of observations were not intended to support multivariable meteorological modelling. These variables were therefore used as contextual information when interpreting between-site and spatial variability rather than as statistically evaluated predictors. Consequently, the present study does not quantify the magnitude or direction of their influence on the measured bacterial concentrations. Future studies specifically designed to investigate environmental determinants of bioaerosol variability should incorporate greater temporal and spatial replication together with continuous meteorological monitoring, enabling multivariable analysis of bacterial concentrations in relation to wind speed, wind direction, temperature, relative humidity, combustion characteristics and other relevant environmental factors.

A further analytical limitation arose from bacterial concentrations exceeding the upper quantification limit of the culture-based method. Four active-burning observations were reported as >1,259 CFU/m^3^, meaning that their true concentrations could not be determined. For exploratory statistical analyses and graphical presentation, these observations were conservatively represented as 1,260 CFU/m^3^. This substitution likely underestimated the magnitude of the highest concentrations and reduced the ability to characterize variability at Sites 1 and 2. Consequently, the reported statistical relationships should be interpreted cautiously. Future studies could employ sampling durations, dilution approaches or complementary enumeration methods capable of resolving higher microbial concentrations and should apply statistical methods appropriate for censored environmental data where necessary.

Culture-dependent analysis imposed additional constraints. TSA incubation at 37 °C under aerobic conditions recovered only microorganisms capable of remaining viable and forming colonies under the selected laboratory conditions. Environmental microorganisms that were viable but non-culturable, required different media or incubation conditions, or were otherwise unable to grow under the applied protocol were not represented (4, 25, 26). The concentrations and taxa reported in the present study therefore represent the culturable bacterial fraction rather than the complete airborne microbial community. Culture-based analysis nevertheless provides complementary information by confirming the recovery of viable organisms capable of growth under the applied conditions. Future studies should combine culture-dependent enumeration with culture-independent molecular approaches, such as 16S rRNA amplicon sequencing or shotgun metagenomics, to characterize broader bacterial diversity and community structure.

Relatedly, the 39 isolates characterized using the Vitek Compact 2 system represent selected cultured isolates rather than a systematically sampled representation of the total bacterial community. Frequencies within this isolate set therefore cannot be interpreted as estimates of taxonomic prevalence, relative abundance, richness or dominance in ambient air. The unresolved Gram-positive bacilli category further limits taxonomic resolution, while biochemical identification using Vitek does not provide the resolution achievable through sequence-based characterization of environmental microorganisms. Accordingly, the isolate data are most appropriately interpreted as demonstrating the types of culturable organisms recovered rather than defining the microbial community associated with informal waste burning. Future investigations should employ predefined and reproducible isolate-selection criteria where culture-dependent identification is retained and complement phenotypic identification with molecular taxonomic methods.

Source attribution was also beyond the scope of the present investigation. Although waste typology, surrounding environmental characteristics and site activities were observed and documented, the study could not distinguish microorganisms originating from waste materials from those derived from soil, vegetation, human activity, background atmospheric communities or other local reservoirs. Nor could it determine the extent to which combustion, thermal convection, mechanical disturbance or wind-driven resuspension contributed to microbial aerosolization. The presence of taxa at both active-burning and comparison locations further demonstrates the difficulty of assigning organisms specifically to waste-burning activity. Future research incorporating microbial source-tracking methods, source-material sampling and appropriate upwind or background air measurements would provide stronger evidence regarding the origins and mobilization pathways of airborne microorganisms in these environments.

The study also did not measure particulate matter, gaseous pollutants or aerosol composition concurrently with bioaerosol sampling. Consequently, although culturable bacteria were detected under visibly active combustion conditions, the study cannot determine whether bacterial concentrations covaried with PM₂.₅, PM₁₀ or gaseous combustion pollutants, or whether recovered microorganisms were physically associated with combustion-derived particles. This distinction is important given the conceptual interpretation of informal waste-burning sites as settings in which chemical and biological exposures may coexist. Future studies should therefore pair bioaerosol sampling with time-resolved measurements of particulate matter and relevant gaseous pollutants to determine whether biological and conventional air-pollution indicators vary concurrently and to investigate potential particle–microorganism associations.

Finally, the study characterized environmental occurrence rather than human exposure or health risk. Sampling within the approximate breathing zone provides information relevant to the potential for inhalation exposure, but no personal monitoring, inhaled dose assessment, virulence testing, clinical outcomes or quantitative microbial risk assessment was undertaken. The detection of viable culturable bacteria, including taxa with recognized opportunistic potential, therefore cannot be interpreted as evidence of infection or adverse health effects. Subsequent research should progress from environmental characterization towards personal exposure assessment and, where justified by stronger exposure evidence, epidemiological or quantitative microbial risk approaches capable of evaluating potential health relevance.

Taken together, these limitations define a clear progression for future research: from the event-based, culture-dependent environmental characterization undertaken here towards longitudinal and highly replicated studies integrating microbial, chemical, meteorological and exposure measurements. Such designs would allow the associations identified in the present exploratory study to be tested more rigorously, distinguish potential microbial sources and mobilization mechanisms, and determine whether the biological component of informal waste-burning environments contributes meaningfully to human exposure.

## 5 Conclusion

This study provides, to our knowledge, the first field-based evidence characterizing culturable airborne bacterial concentrations within active informal waste-burning environments in a South African peri-urban setting. Culturable bacterial concentrations were generally higher at active-burning sites than at the non-burning comparison site, with several observations at Sites 1 and 2 exceeding the analytical upper quantification limit (>1,259 CFU/m^3^). However, concentrations at Site 3 overlapped with those of the comparison environment, demonstrating that elevated bacterial occurrence was not uniform across all active-burning locations. These findings establish that viable culturable bacteria occur within the airborne environment associated with active informal waste burning and identify a biological dimension of these settings that has received comparatively little attention alongside their well-established chemical-pollution burden.

Beyond documenting bacterial concentrations, the study demonstrates substantial spatial and between-site variability in culturable bacterial occurrence. No consistent distance-dependent gradient was observed across the active-burning sites, indicating that distance from the combustion area alone was insufficient to characterize spatial patterns in bacterial concentrations. Field observations identified differences in combustion intensity, smoke-plume behavior, waste composition, meteorological conditions and physical disturbance that provide plausible context for this variability, although their independent contributions were not quantified. Informal waste-burning sites should therefore be understood as dynamic environmental settings in which multiple potential microbial reservoirs and aerosolization and transport processes coexist.

The selected culturable isolates further illustrated the range of bacterial taxa recoverable from these environments, including environmentally widespread organisms and taxa also reported from human-, animal- and waste-associated settings. The occurrence of several identified taxa at both active-burning and comparison sites indicates that the recovered organisms were not unique to waste-burning locations. However, because identification was conducted on selected cultured isolates, the study does not establish community-level taxonomic composition, relative abundance or a combustion-specific microbiome. Similarly, the present findings do not establish that combustion itself generated the bacteria detected or determine their specific environmental sources. Rather, heterogeneous waste materials, soil, vegetation, anthropogenic activity and surrounding environmental reservoirs represent multiple plausible sources from which microorganisms may become airborne.

From an environmental-health perspective, the findings support conceptualizing informal waste-burning environments as potential mixed chemical-biological exposure settings rather than solely as sources of conventional chemical pollution. The study did not concurrently measure chemical pollutants, personal microbial exposure, inhaled dose, pathogenicity or health outcomes and therefore does not establish human health risk or disease causation. Instead, it establishes environmental occurrence: viable culturable bacteria were present in air sampled during active informal waste-burning conditions. This evidence provides a basis for investigating whether and under what conditions such biological material contributes meaningfully to human exposure, particularly where recurrent burning occurs near residential and other sensitive environments.

Future research should build on these findings through longitudinal and seasonally replicated sampling, greater spatial and temporal resolution, concurrent measurement of particulate and gaseous pollutants, continuous meteorological monitoring and molecular microbial characterization. Such designs would enable multivariable evaluation of the relative contributions of meteorology, combustion characteristics, waste composition and physical disturbance, while source-tracking and personal-exposure approaches could clarify microbial origins and potential human exposure. Importantly, the present findings do not justify immediate microbial air-quality standards or routine regulatory monitoring. Rather, they demonstrate the value of integrating environmental microbiology with atmospheric science, waste management and exposure assessment when investigating informal waste-burning environments.

Ultimately, the principal contribution of this study is the identification of viable culturable bacteria as an under-characterized component of the airborne environment associated with informal waste burning. By extending investigation beyond conventional chemical pollutants while maintaining clear boundaries between environmental occurrence, source attribution, exposure and health risk, the study provides a foundation for more integrated investigation of informal waste burning as a complex environmental and public-health challenge in peri-urban communities.

## Data Availability

The original contributions presented in the study are included in the article/supplementary material, further inquiries can be directed to the corresponding author.
